# Distribution of soil nutrients and erodibility factor under different soil types in an erosion region of Southeast China

**DOI:** 10.7717/peerj.11630

**Published:** 2021-06-16

**Authors:** Man Liu, Guilin Han

**Affiliations:** Institute of Earth Sciences, China University of Geosciences (Beijing), Beijing, China

**Keywords:** Soil organic carbon and nitrogen, Soil major elements, Soil erodibility, Alfisols, Southeast China

## Abstract

**Background:**

Soil erosion can affect the distribution of soil nutrients, which restricts soil productivity. However, it is still a challenge to understand the response of soil nutrients to erosion under different soil types.

**Methods:**

The distribution of soil nutrients, including soil organic carbon (SOC), soil organic nitrogen (SON), and soil major elements (expressed as Al_2_O_3_, CaO, Fe_2_O_3_, K_2_O, Na_2_O, MgO, TiO_2_, and SiO_2_), were analyzed in the profiles from yellow soils, red soils, and lateritic red soils in an erosion region of Southeast China. Soil erodibility K factor calculated on the Erosion Productivity Impact Calculator (EPIC) model was used to indicate erosion risk of surface soils (0∼30 cm depth). The relationships between these soil properties were explored by Spearman’s rank correlation analysis, further to determine the factors that affected the distribution of SOC, SON, and soil major elements under different soil types.

**Results:**

The K factors in the red soils were significantly lower than those in the yellow soils and significantly higher than those in the lateritic red soils. The SON concentrations in the deep layer of the yellow soils were twice larger than those in the red soils and lateritic red soils, while the SOC concentrations between them were not significantly different. The concentrations of most major elements, except Al_2_O_3_ and SiO_2_, in the yellow soils, were significantly larger than those in the red soils and lateritic red soils. Moreover, the concentrations of major metal elements positively correlated with silt proportions and SiO_2_ concentrations positively correlated with sand proportions at the 0∼80 cm depth in the yellow soils. Soil major elements depended on both soil evolution and soil erosion in the surface layer of yellow soils. In the yellow soils below the 80 cm depth, soil pH positively correlated with K_2_O, Na_2_O, and CaO concentrations, while negatively correlated with Fe_2_O_3_ concentrations, which was controlled by the processes of soil evolution. The concentrations of soil major elements did not significantly correlate with soil pH or particle distribution in the red soils and lateritic red soils, likely associated with intricate factors.

**Conclusions:**

These results suggest that soil nutrients and soil erodibility K factor in the yellow soils were higher than those in the lateritic red soils and red soils. The distribution of soil nutrients is controlled by soil erosion and soil evolution in the erosion region of Southeast China.

## Introduction

Soil erosion is considered the most serious environmental problem of soil degradation, which threatens local food security, eco-environment, and social sustainability (Quinton et al., 2010; Zhang et al., 2018). Water erosion is the predominant form of soil erosion affecting about 1094 million ha, compared to wind erosion, which affected 549 million ha of land globally (Lal, 2003). In China, although soil erosion has been ameliorated since the implementation of the ‘Grain for Green Project’ in the 1990s, 129 million ha of land was still affected by soil erosion (Zhang et al., 2019). Basin is the base research unit of water erosion, for example, previous research has mainly focused on the Yangtze River basin (Fan et al., 2010; Fang et al., 2019; Ma et al., 2003), Yellow River basin (Ni, Li & Borthwick, 2008; Ouyang et al., 2010; Wang et al., 2018; Zhao et al., 2018), and Pearl River basin (Hu, Wu & Li, 2019; Lai et al., 2016; Liu et al., 2015; Wei & Wang, 2006). Although the erosion area of Fujian province has decreased in recent years, about 9521 km^2^ (accounting for 7.75% area of Fujian province, MWRPRC, 2019) lands were still threatened by soil erosion. The Jiulongjiang River basin in Fujian province is located in the region with a subtropical monsoon climate, which is characteristic of adequate rainfall and long precipitation period (Zhang et al., 2012). Intensive and continuous rainfall increases the threat of soil loss, specifically in the multi-sloped hilly region. Although the forest coverage rate is high (Quan et al., 2007), the shortage of grass and litter layers under the canopy leads to a lower capacity of water retention (Zhang, DeAngelis & Zhuang, 2011a). Human activities, including agricultural reclamation and architectural excavation, destroy soil structure, resulting in increased soil erosion (Liu, Han & Li, 2021a). In recent years, the effectiveness of water and soil conservation measures has been far weaker than the loss caused by soil erosion (Huang, 2016). Therefore, it is urgent to enhance the basic understanding of soil erosion patterns, which is helpful to effectively control soil erosion in the red soil region.

In addition to the research of basic mechanisms about soil erosion patterns, the response of soil nutrients to erosion is also widely concerned (Chamizo et al., 2017; Pimentel & Burgess, 2013; Quinton et al., 2010; Zhu, Deng & Shangguan, 2018; Hu et al., 2020). Soil erosion is considered one of the driving forces that affect the dynamics of soil nutrients (Borrelli et al., 2017). Soil nutrients, including soil major elements, soil organic nitrogen (SON), and soil organic carbon (SOC), are regarded as the key indexes of soil fertility. Generally, soil erosion causes the redistribution of soil nutrients. Soil erosion can also affect the transformation of these nutrients (Ge et al., 2007), for example, SOC mineralization (Wang et al., 2020), soil N transformation (Piao et al., 2020; Yue et al., 2020), and dissolution and precipitation of soil metal elements (Shi et al., 2020). The effect of soil erosion on SOC dynamics is widely understood in terms of the significance of the global C cycle (Lal, 2003), but the influence on soil fertility is also non-negligible (Liu & Han, 2021; Zhang et al., 2013). Zhu, Deng & Shangguan (2018) suggested that soil erosion promoted the conversion of organic N into inorganic N. Li et al. (2017) considered that long-term cultivation reduced soil Ca^2+^ concentration under erosion, which affected SOC stabilization through the combination of clay minerals and organic matters. In the red soil region of southeastern China, the problem of soil health has been frequently underscored due to the extremely low level of soil nutrients (Zhang et al., 2013). Generally, the concentrations of soil nutrients in the yellow soils are higher than those in the red soils and lateritic red soils. It is closely linked with the different degrees of weathering and leaching (Quan et al., 2007). However, it is hard to distinguish the iteration effects of weathering and leaching processes and erosion processes on the loss of soil nutrients in the erosion region. The erosion risk of surface soils is indicated by soil erodibility, which is generally measured by the K factor of the Universal Soil Loss Equation (USLE) model (Wischmeier & Smith, 1965).

Thus, research about the distribution of soil nutrients and soil erodibility K factor across different soil types (including yellow soils, red soils, and lateritic red soils) is conducive to understanding the loss mechanism of soil nutrients under the erodible environment. We hypothesized that both soil erosion and soil evolution control the distribution of soil nutrients in the erosion region of Southeast China. This study is closely associated with the conservation of soil nutrients, which is critical for ecological stability (Gong et al., 2020). Therefore, the objects of the study were: (1) to analyze the variations of soil erodibility K factor in surface soils and the concentrations of SON, SOC, and soil major elements in the soil profiles under different soil types; (2) to determine the factors that affect the distribution of SOC, SON and soil major elements, and soil erodibility K factor under the erosion region of Southeast China.

## Material and Methods

### Study area

**Figure 1:**
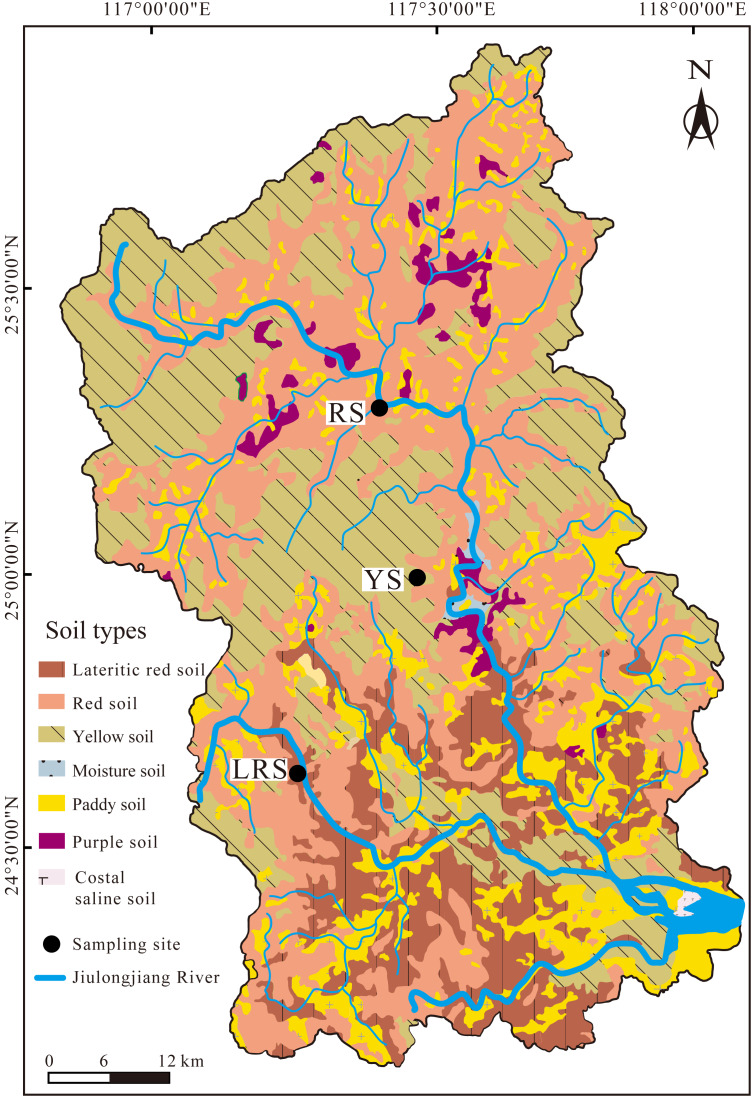
The distribution of soil types in the Jiulongjiang River basin and location of sampling sites.

The study area is located in the red soil region of Southeast China, in which the Jiulongjiang River basin is one of the typical erosion regions. Thus, the Jiulongjiang River basin was selected as the sampling region. The Jiulongjiang River basin (24°18′−25°88′N, 116°78′−118°03′E) is located in Fujian Province, with a drainage area of 14,741 km^2^ (Fig. 1). The average elevation is less than 200 m above sea level. The terrain of the basin decreases from north to south, transits from mountain to plain. The basin is controlled by the subtropical and tropical monsoon climate, annual precipitation is about 1,400∼1,800 mm and annual temperature is about 19.9∼21.1 °C (Zhang et al., 2012). The original forest accounts for over 60% of the basin area, other lands are mainly used for agriculture and residential area. This area distributes yellow soils, red soils, lateritic red soils, purple soils, and paddy soils (Fig. 1). Over 90% of the basin area is covered by red soils, lateritic red soils, and yellow soils. Moreover, most soils are developed from granites and clastic rocks (Quan et al., 2007). These soils can be classified into the Alfisols based on the soil taxonomy of the United States Department of Agriculture (USDA) (Soil Survey Staff, 2014).

### Soil sampling

Soil samples were collected in the winter season of January 2018. The three soil sites were selected according to different soil types, including red soils, lateritic red soils, and yellow soils, respectively (Fig. 1). The soil profiles were located at the excavation ground, furthermore, the sampling work was obtained support and help from the person in charge of the construction company. Considering the strong spatial heterogeneity of soils, especially in the vertical direction, the averaged results from the parallel soil profiles (if the distance between them is extremely far) will lose, even mislead the information that individual soil profile carries. Thus, the distance between the three parallel soil profiles at the same site is less than one m. A total of 9 soil profiles with a thickness of three m were used to collect soil samples. Soil samples were orderly collected from the bottom to the top to avoid pollution between samples, with a five cm-interval. The three samples at the same depth derived from the three parallel soil profiles were mixed to be one sample. In total, there are 180 samples. The replication of sampling sites and soil samples were considered together in the present study. The thickness and visible characteristics of the three soil profiles are shown in Table 1.

**Table 1:** Location, land use type, and prole description of sampling sites.

Sampling site	Longitude and latitude	Elevation (m)	Soil Types	Slope aspect and slope gradient	Visible characteristic of soil profile
YS	117°30′9.49″E; 24°59′39.7″N	128	Yellow soil	South-facing slope; < 5°	0–30 cm: Dark brown humus layer, fine sand, loose; 30–50 cm: Gray mixed yellow, fine sand, loose; 50–300 cm: Yellow, clay-grained, tight.
LRS	117°14′5.16″E; 24°39′6.33″N	131	Lateritic red soil	South-facing slope; < 5°	0–40 cm: Dark brown humus layer, fine sand mixed few coarse sand, loose; 40–70 cm: Gray, fine sand mixed few coarse sand, loose; 70–300 cm: Red, fine sand mixed few coarse sand, loose.
RS	117°25′28.11″E; 25°16′21.08″N	221	Red soil	South-facing slope; < 5°	0–70 cm: Dark red humus layer, fine-grained, loose; many fine roots 70–300 cm: Red, fine-grained, loose.

### Sample analysis

Soil samples were air-dried after removing gravel and fresh roots, and then were preserved after passing through a two mm sifter. Soil pH values were determined by a pH meter (Liu, Han & Li, 2021a). Soil particle distributions were determined on a laser particle size analyzer (Liu, Han & Zhang, 2020). Soil samples (<2 mm) were ground into powder (<75 µm), using for further analysis of the concentrations of soil major elements. The soil samples were digested with three mL HF, one mL HClO_4_, and three mL HNO_3_ at 120 °C for 3 days (Li et al., 2020). The concentrations of soil major elements were expressed as the concentrations of their oxides (i.e., Al_2_O_3_, CaO, Fe_2_O_3_, K_2_O, Na_2_O, MgO, and TiO_2_, %) in the soils (Liu, Han & Li, 2021a). The SiO_2_ concentrations were analyzed according to Du et al. (2014).

Carbonates in soil samples (<75 µm) were removed by treating with 0.5 mol L^−1^ HCl for 24 h (Midwood & Boutton, 1998), and inorganic N were removed by treating with 2 mol L^−1^ KCl for 24 h (Meng, Ding & Cai, 2005). The treated samples were washed with purified water until neutrality, then were dried at 55 °C until constant weight and ground into powder. The SOC and SON concentrations were measured by a multi-element analyzer. Actual SON and SOC concentrations in the original soils can be calibrated by multiplying of measured value by the ratio of the sample mass after treating to it before treating.

### Calculation of the K factor on the EPIC model

Soil erodibility can be used to indicate the erosion risk of surface soils because of the significant quantitative relationship between the amount of soil loss and the K factor (Wischmeier & Smith, 1965). Many modified models have been proposed based on correlations between soil physicochemical properties and the K values (Wang et al., 2013). For example, the K factor of the Erosion Productivity Impact Calculator (EPIC) model (Sharpley & Williams, 1990) is calculated using the parameters, including SOC concentration and different-sized particle distribution. Soil structure affects soil erodibility by restricting the force of slope flow. Many researchers reported that water-stable macro-aggregates could decrease soil erodibility due to the effects on soil structure (Ding & Zhang, 2016; Liu & Han, 2020). However, the formation and stabilization of water-stable aggregates are closely associated with the rich soil organic matter (SOM) (Six & Paustian, 2014). Water-stable macro-aggregates were hardly formed in the SOM-poor red soils of Southeast China, based on field observation. Therefore, SOC concentration and different-sized particle distribution are the main factors that affect soil erodibility. Thus, the EPIC model is fit to estimate soil erodibility K factor in the study area, and the formula is shown as follow:

\begin{eqnarray*}\text{Kepic}& = \left\{ 0.2+0.3\exp \nolimits \left[ -0.0256\mathrm{Sa} \left( 1- \frac{\mathrm{Si}}{100} \right) \right] \right\} \times { \left( \frac{\mathrm{Si}}{\mathrm{Si}+\mathrm{Cl}} \right) }^{0.3}\nonumber\\\displaystyle & \quad  \times \left[ 1.0- \frac{0.25\mathrm{C}}{\mathrm{C}+\exp \nolimits \left( 3.72-2.95\mathrm{C} \right) } \right] \times \left[ 1.0- \frac{0.7\mathrm{SN}1}{\mathrm{SN}1+\exp \nolimits \left( -5.51+22.9\mathrm{SN}1 \right) } \right] \end{eqnarray*}

where the Sa, Si, and Cl (%) are the proportions of sand-sized, silt-sized, and clay-sized particles, respectively; the SN1 (%) = 1–Sa/100; the C (%) is SOC concentration. The unit of the K factor is *t acre h/100 acre/ft/tanf/in*. For brevity, the unit of the K factor will not be noted.

### Statistical analysis

Boxplot was used to show the ranges of the proportions of different-sized particles, soil pH values, the concentrations of SOC, SON, and soil major elements (expressed as Al_2_O_3_, CaO, Fe_2_O_3_, K_2_O, Na_2_O, MgO, TiO_2_, and SiO_2_), and the K factor values in the red soils, lateritic red soils, and yellow soils. One-way ANOVA with the least significant difference (LSD) test was performed to determine the differences in the proportions of different-sized particles, soil pH values, the concentrations of SOC, SON, and soil major elements, and the K factor values among the three soil types at the level of *P* < 0.05. All data were calculated via the Kolmogorov–Smirnov test, which is one of the non-parametric tests commonly applied to analyze the normal distribution of the sample data set (Zeng & Han, 2020). Spearman’s rank correlation coefficient determined the relationship between different soil properties. All statistical analyses were performed by the SPSS 18.0 software (SPSS Inc., Chicago, IL, USA) and all graphs were drawn by SigmaPlot 12.5 software (Systat Software GmbH, Erkrath, Germany).

## Results

### Soil particle distribution and soil pH

**Figure 2:**
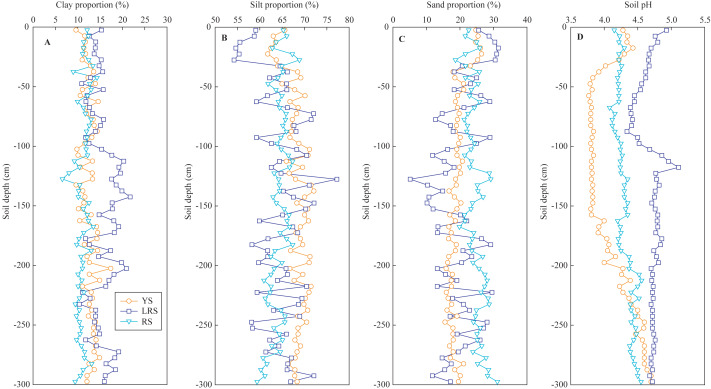
The vertical distributions of clay proportion (A), silt proportion (B), sand proportion (C) and soil pH (D) in soil profiles. YS, yellow soil; LRS, Lateritic red soil; RS, red soil.

**Figure 3:**
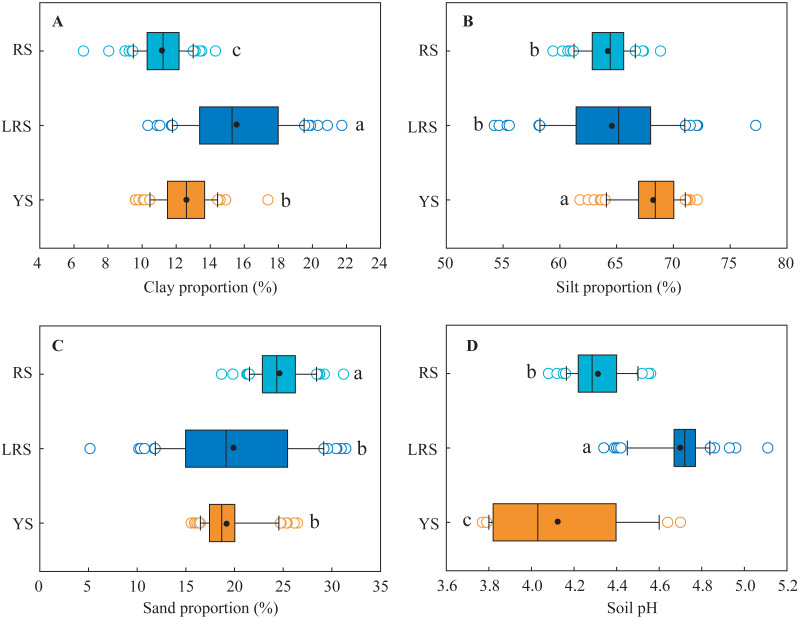
The ranges of clay proportion (A), silt proportion (B), sand proportion (C) and soil pH (D) in different soil profiles. Lowercases indicate significant differences in the proportion of different-sized particles and soil pH between different profiles at the threshold of *P* < 0.05 level, based on the least significant difference (LSD) test. YS, yellow soil; LRS, lateritic red soil; RS, red soil.

The proportions of different-sized particles and soil pH in the three profiles are shown in Figs. 2 and 3, respectively. The clay proportions in the YS and RS profiles were almost constant with increasing soil depth, while silt proportions increased in the YS profile and decreased in the RS profile, and sand proportions decreased in the YS profile and increased in the RS profile (Fig. 2). The vertical distributions of the different-sized particles in the LRS profile showed intensive fluctuation, which was attributed to the non-uniform distribution of the large-sized unweathered quartz particles. On the whole, the clay proportions in the yellow soils (mean: 13%) were significantly lower than those in the lateritic red soils (mean: 15%) and were significantly larger than those in the red soils (mean: 11%) (Fig. 3). The silt proportions in the yellow soils (mean: 68%) were significantly larger than those of the lateritic red soils (mean: 64%) and red soils (mean: 64%). While the sand proportions in the yellow soils (mean: 19%) were significantly lower than those in the red soils (mean: 25%) and were similar to those in the lateritic red soils (mean: 20%). The texture of most soils in the three profiles belongs to silt loams (Soil Survey Staff, 2014).

The soil pH values in the YS profile decreased from 4.3 to 3.8 with increasing soil depth at the 0∼50 cm depth, then kept constant at the 50∼160 cm depth, subsequently increased to 4.7 at the bottom (Fig. 2). In the RS profile, the soil pH values increased from 4.2 to 4.6 with increasing soil depth. In the LRS profile, the soil pH values decreased from 4.9 to 4.3 at the 0∼90 cm depth, then increased to 5.1 at the 120 cm depth, and were constant (4.7) in the deeper soils. On the whole, the soil pH values in red soils (mean: 4.3) were significantly lower than those in the lateritic red soils (mean: 4.7) and were significantly larger than those in the yellow soils (mean: 4.1) (Fig. 3). All soils in the three profiles were strong acid.

### SOC and SON concentration and C/N ratio

**Figure 4:**
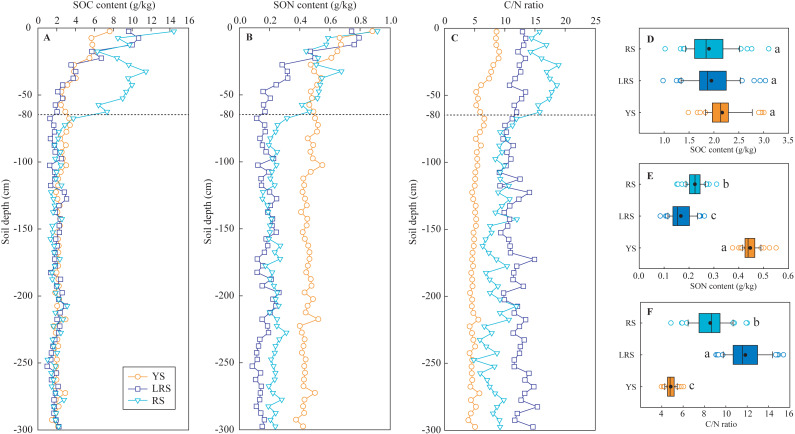
The vertical variations of SOC content (A), SON content (B), and C/N ratio (C) in soil profiles and their ranges in the soils below the 80 cm depth (D, E, F). Lowercases in the D, E and F figures indicate significant differences in SOC content, SON content, and C/N ratio between the different profiles (below the 80 cm depth) at the threshold of *P* < 0.05 level, based on the least significant difference (LSD) test. YS, yellow soil; LRS, lateritic red soil; RS, red soil.

The concentrations of SOC and SON in the three profiles showed decreasing trends with increasing soil depth at the 0∼80 cm depth, while they were almost constant in the soils below the 80 cm depth (Fig. 4). In the soils at the 0∼80 cm depth, the SOC and SON concentrations in the YS profile decreased from 8 g/kg to 2 g/kg and from 0.9 g/kg to 0.4 g/kg with increasing soil depth, respectively. Similarly, the SOC concentrations in the LRS profile decreased from 11 g/kg to 2 g/kg, and the SON concentrations decreased from 0.8 g/kg to 0.2 g/kg. However, the SOC and SON concentrations in the RS profile showed abnormal increasing trends at the depth of 20∼70 cm, which was closely associated with the abundant plant fine root (Table 1). In the soils below the 80 cm depth, the SOC concentrations in the three profiles were not significantly different, while the SON concentrations in the YS profile were significantly lower than those in the LRS and RS profiles.

The C/N ratios in the YS and LRS profiles slowly decreased from 8.6 to 4.0 and from 13.4 to 11.6 with increasing soil depth, respectively. However, the C/N ratios in the RS profile intensively decreased at the 80 cm depth. For the soils below the 80 cm depth, the C/N ratios in the YS profile were significantly lower than those in the LRS and RS profiles.

### Soil major elements

**Figure 5:**
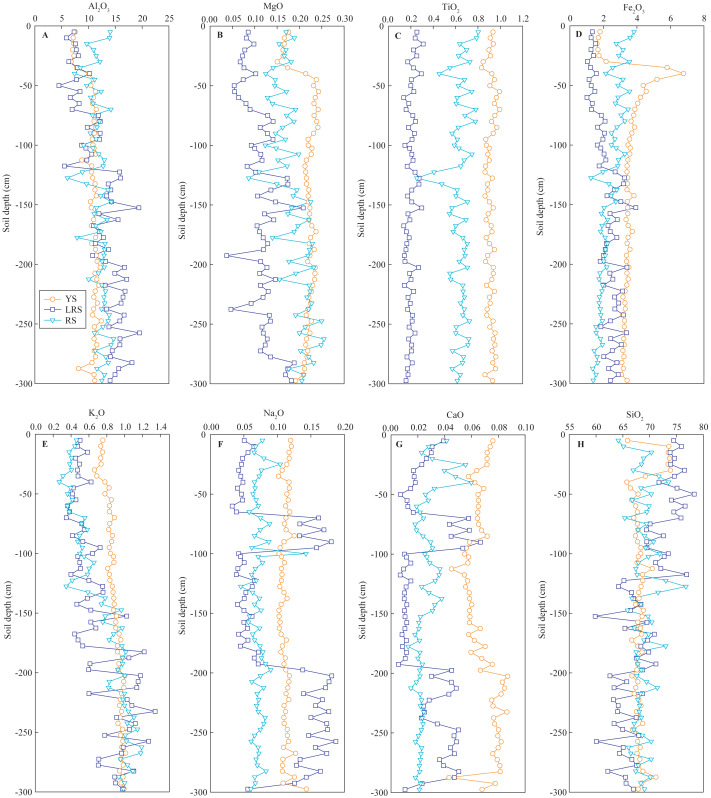
The vertical variations of the content of soil major elements in soil profiles. The results are expressed in the contents of the oxides, including Al_2_O_3_ (A), MgO (B), TiO_2_ (C), Fe_2_O_3_ (D), K_2_O (E), Na_2_O (F), CaO (G) and SiO_2_ (H). YS, yellow soil; LRS, lateritic red soil; RS, red soil.

**Figure 6:**
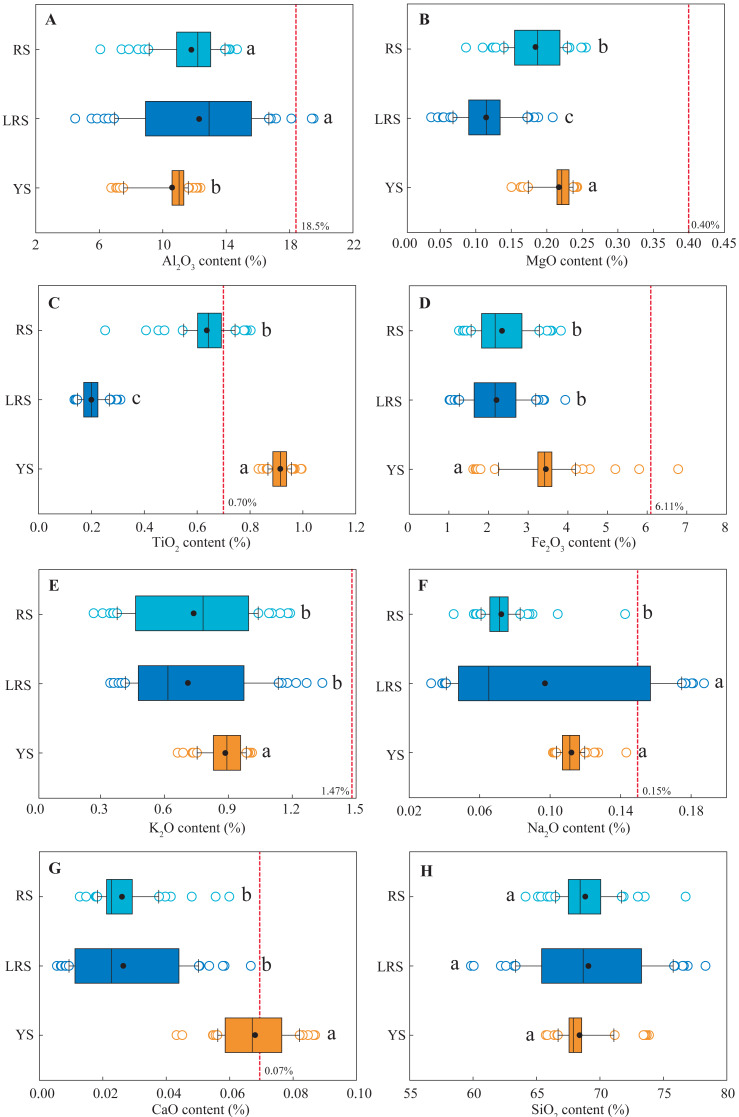
The ranges of the content of soil major elements in different soil profiles. The results are expressed in the contents of the oxides, including Al_2_O_3_ (A), MgO (B), TiO_2_ (C), Fe_2_O_3_ (D), K_2_O (E), Na_2_O (F), CaO (G) and SiO_2_ (H). Lowercases indicate significant differences in the content of soil major elements between different profiles at the threshold of *P* < 0.05 level, based on the least significant difference (LSD) test. The red dotted lines indicate the background values of soil major elements in Fujian Province (Chen et al., 1992). YS, yellow soil; LRS, lateritic red soil; RS, red soil.

The vertical distributions of soil major elements, including Al_2_O_3_, MgO, TiO_2_, Fe_2_O_3_, K_2_O, Na_2_O, CaO, and SiO_2_ in the three soil profiles are shown in Fig. 5. In the YS profiles, the concentrations of Al_2_O_3_, MgO, and Fe_2_O_3_ in the surface soils were significantly lower than those in the deep soils. The K_2_O and CaO concentrations slightly increased with increasing soil depth, while the concentrations of TiO_2_, Na_2_O, and SiO_2_ were almost constant, except for the abnormally high SiO_2_ concentrations that occurred in the layer of the 5∼35 cm depth. The distributions of these soil major elements were fluctuant in the LRS and RS profiles. Generally, the MgO and K_2_O concentrations in the RS profile slightly increased with increasing soil depth, while Fe_2_O_3_ and CaO concentrations slightly decreased. In the LRS profile, the Al_2_O_3_, MgO, Fe_2_O_3_, and K_2_O concentrations increased and SiO_2_ decreased with increasing soil depth. Particularly, the Na_2_O and CaO concentrations at the 70∼90 cm and 200∼295 cm depth occurred abnormal peaks. On the whole, the Al_2_O_3_ concentrations in the YS profile were significantly lower than those in the RS and LRS profiles, while the MgO, TiO_2_, Fe_2_O_3_, K_2_O, Na_2_O, and CaO were significantly larger (Fig. 6). The MgO and TiO_2_ concentrations in the LRS profile were significantly lower than those in the RS profile, and the Na_2_O were significantly lower, while the Al_2_O_3_, Fe_2_O_3_, K_2_O, CaO concentrations in the two profiles were not significantly different. The SiO_2_ concentrations in the three soils were similar with a mean proportion of 68%. Compared to the background values of soil major elements in Fujian Province (Chen et al., 1992), except for TiO_2_ and CaO in the YS profile, the major elements in the three profiles were almost lower than these background values.

### Soil erodibility K factor

**Figure 7:**
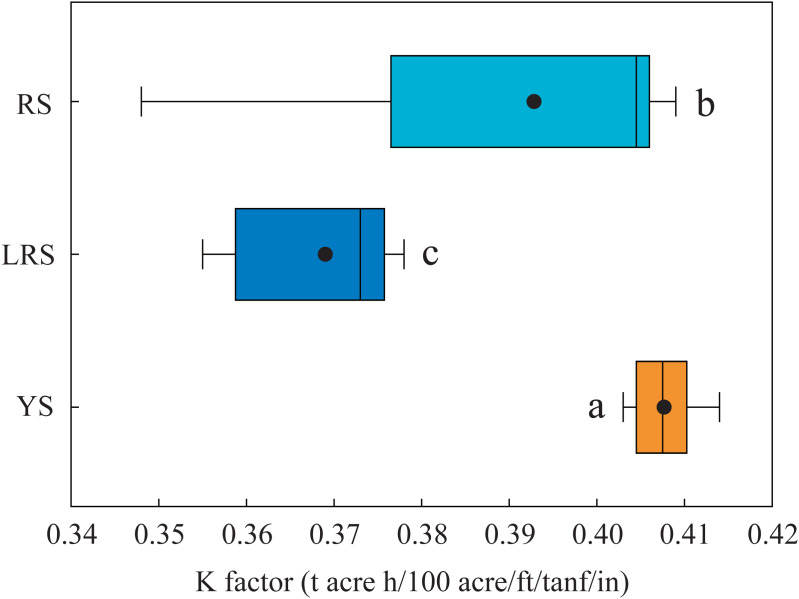
The K factor values in the soils at 0∼30 cm depth of the three soil profiles. Lowercases indicate significant differences in the K factor between different the soil profiles (below the 80 cm depth) at the threshold of *P* < 0.05 level, based on the least significant difference (LSD) test. YS, yellow soil; LRS, lateritic red soil; RS, red soil.

The K factors in the soils at the 0∼30 cm depth of the three profiles ranged from 0.348 to 0.414 (Fig. 7). The K factors in the red soils (mean: 0.393) were significantly lower than those in the yellow soils (mean: 0.407) and significantly higher than those in the lateritic red soils (mean: 0.369).

## Discussion

### Soil erodibility K factor in different soils

Soil erodibility K factor on the EPIC model is affected by soil physicochemical properties, including SOC concentration and the proportions of different-sized particles (Sharpley & Williams, 1990). Soil structure significantly affects the erosive force of runoff (Liu & Han, 2020). In the study area, SOM abundance and the distribution of different-sized particles mainly determined soil structure. SOM plays an important role in affecting soil erodibility, due to the ability to absorb water and enhance soil infiltration (Zhu et al., 2010). The distribution of soil particles determines soil porosity, which is also key for soil infiltration. Although water-stable aggregates have been widely reported that can effectively enhance soil structure (Ding & Zhang, 2016; Liu & Han, 2020). However, the low SOC concentration in the yellow soils, lateritic red soils, and red soils restricted the formation of water-stable aggregates. Thus, the K factor estimated on the EPIC model can be sufficiently used to predict erosion risk in the red soil region. The K factor values (0.348∼0.414, Fig. 7) in this study were accordant with the range of 0.15∼0.45 in the surface soils (at the 0∼30 cm depth) of Southern China (Zhang, DeAngelis & Zhuang, 2011b). Undoubtedly, the exact values were determined by the SOC concentration and the proportions of different-sized particles in soils. The K factors in the yellow soils were significantly higher than those in the red soils, and those in the red soils significantly higher than those in the lateritic red soils (Fig. 7). This result showed that soil erodibility significantly decreased with the progress of soil evolution. Under the effects of weathering and leaching processes, the progress of soil evolution generally follows as yellow soils, red soils, and lateritic red soils in Southeast China (Quan et al., 2007). In the soil evolution process, soil texture trends more and more clayey (Quan et al., 2007). Soil crust is easily formed in the clayed soils to reduce the loss of soil particles (Wang et al., 2018). Thus, the soil erodibility K factors of the three soils were significantly different.

### Soil organic carbon and nitrogen in different soils

The organic carbon level in soils depends on the balance between the plant input and output due to microbial decomposition and soil erosion (Han et al., 2020; Jobbagy & Jackson, 2000; Yu et al., 2020). Deciduous forests are widely distributed in the red soil hilly area of southern China, which provides sufficient organic matter input. Generally, the low SOC concentration in the red soil region is mainly attributed to the rapid SOC turnover (Chen, He & Huang, 2002). Firstly, the warm humid monsoon climate in this region is conducive to microbial activities, resulting in the rapid decomposition of organic matter (Goh, 2011; Xia et al., 2021). Secondly, organic matters are lost as dissolved humic acid under soil erosion. The accumulation of humic acid generally leads to the decreasing soil pH, thus it is commonly observed that soil pH increases with increasing soil depth. However, the decreasing trend of soil pH with increasing soil depth in the YS and LRS profiles at the 0∼80 cm depth (Fig. 2), which mainly is attributed to the loss of dissolved humic acid. Thirdly, lots of Ca^2+^ and Mg^2+^ are lost in the processes of red soil evolution and leaching, resulting in reduced SOC stabilization. Soil Ca^2+^ and Mg^2+^ play important roles in concatenating organic matters with clay minerals to form stable organic–inorganic complexes (Li et al., 2017). The shortage of Ca^2+^ and Mg^2+^ can cause the acceleration of SOC decomposition due to the decreased stability. Additionally, it has been widely reported that the loss of particle organic matter causes decreased SOC concentration in surface soils (Ge et al., 2007; Quinton et al., 2010; Zhang et al., 2018). Thus, the soil erosion and soil physicochemical properties (depended on soil evolution) affect SOC distribution.

The SON concentrations in the yellow soils below the 80 cm depth were significantly larger (twice) than those in the red soils and lateritic red soils (Fig. 4). The large SON concentrations in the deep layer of the yellow soils were mainly attributed to the re-assimilation of inorganic N through microbial immobilization. This conclusion can be inferred by the results of soil C/N ratios and *δ*^15^N values (Table S1). The C/N ratio of plant-derived organic matter generally decreases in the processes of microbial decomposition (Marshall, Brooks & Lajtha, 2007), but it is not lower than 5 of the mean C/N ratio of microbial biomass (Zhang et al., 2000). The C/N ratios in the yellow soils below the 80 cm depth were almost near 5, thus it can be confirmed that the most SON is derived microbial biomass or necromass. The mean *δ*^15^N value of SON in the deep soils was 3‰, which was ^15^N-depleted compared to the decomposed organic matter (5‰) in the upper layer. It is can be inferred that the re-assimilation of ^15^N-depleted inorganic N through microbial immobilization into microbial biomass (Boutton & Liao, 2010; Corre et al., 2007), resulting in the ^15^N-depleted SON in the deep soils. In agricultural ecosystems, excess inorganic N generally is assimilated by soil microbes in the deep soils (Baggs et al., 2003; Liu, Han & Li, 2021b).

### Soil major elements in different soils

Compared to the background values of soil major elements in Fujian Province (Chen et al., 1992), the major elements in the three profiles were almost lower than the background values (Fig. 5). The dissolved metal ion is easily lost under the strong erosion environment. However, the concentrations of many major elements in the yellow soils with a high erodibility were significantly larger than those in the red soils and lateritic red soils (Fig. 6). The result indicated that soil evolution significantly affected the distribution of soil major elements under different soil types. In the region of Southeast China, soil Na^+^, K^+^, Ca^2+^, and Mg^2+^ are preferentially dissolved and lost under strong leaching conditions, while the iron and aluminum, these have a low mobilization, are gradually accumulated with soil evolution (Belton & Goh, 1992; Treder, 2005). Furthermore, soil pH generally decreases with increasing soil depth because H^+^ replaces the position of the positive charge. Thus, theoretically, soil pH positively correlates with K_2_O, Na_2_O, CaO, and MgO concentrations, while negatively correlates with Al_2_O_3_, TiO_2_, and Fe_2_O_3_ concentrations. In the yellow soils, similar correlations between soil pH and the concentrations of major metal elements were observed below 80 cm depth (Table 2), indicating that the distribution of these elements in the deep soils is controlled by soil evolution. However, soil pH negatively correlated with the concentrations of all major metal elements and positively correlated with SiO_2_ concentration at 0∼80 cm depth (Table 2). This result indicated that the distribution of the major elements was also controlled by other factors. Silt proportion positively correlated with the concentrations of all major metal elements and negatively correlated with SiO_2_ concentration; whereas sand proportion negatively correlated with the concentrations of all major metal elements and positively correlated with SiO_2_ concentration (Table 2). These results indicated that the distribution of soil major elements in the surface soils was closely associated with the distribution of soil particles. Soil erosion preferentially removes fine particles (Ostovari et al., 2018). Thus, the variation of soil major elements in the surface layer of the yellow soils was affected by both soil erosion and soil evolution. The concentrations of most major elements did not significantly correlate with soil pH or the proportion of different-sized particles in the red soils and lateritic red soils, likely associated with intricate factors, for examples, extremely low concentration of some elements, strong leaching, and differences in soil parent materials. More studies are needed to explore the reasons that cause the vertical variations of soil major elements in different soil types.

**Table 2:** Spearman’s rank correlation coecients between the proportion of dierent-sized particles, soil pH and oxide contents in the soils at the 0∼80 cm depth and in the soils below the 80 cm depth of the three soil proles.

	Al_2_O_3_	MgO	TiO_2_	Fe_2_O_3_	K_2_O	Na_2_O	CaO	SiO_2_
The soils at 0∼80 cm depth in the YS profile								
Clay	0.28	0.23	−0.08	0.46	0.19	−0.16	−0.40	−0.13
Silt	0.81**	0.86**	0.75**	0.67**	0.81**	−0.04	−0.43	−0.80**
Sand	−0.71**	−0.77**	−0.59*	−0.72**	−0.72**	−0.06	0.49	0.71**
Soil pH	−0.89**	−0.86**	−0.63**	−0.66**	−0.82**	0.28	0.51*	0.55*
The soils below 80 cm depth in the YS profile								
Clay	0.26	0.26	0.53**	0.02	0.44**	0.35*	0.33*	−0.29
Silt	0.27	0.10	−0.30*	−0.08	−0.05	−0.14	−0.02	−0.20
Sand	−0.62**	−0.38*	−0.22	0.14	−0.51**	−0.28	−0.46**	0.55**
Soil pH	0.20	−0.22	0.35*	−0.76**	0.83**	0.49**	0.71**	−0.04
The soils at 0∼80 cm depth in the LRS profile								
Clay	0.50*	0.30	0.56*	0.44	0.68**	0.34	0.25	−0.59*
Silt	0.49*	0.34	−0.08	0.47	0.05	0.41	0.16	−0.33
Sand	−0.50	−0.34	−0.06	−0.47	−0.23	−0.42	−0.14	0.37
Soil pH	−0.46	−0.14	0.48	−0.25	0.30	−0.11	−0.03	0.18
The soils below 80 cm depth in the LRS profile								
Clay	−0.07	0.00	0.08	−0.02	−0.37*	−0.39**	−0.26	0.08
Silt	0.10	0.16	0.16	0.28	−0.12	−0.13	−0.12	−0.12
Sand	−0.03	−0.18	−0.19	−0.21	0.29	0.32*	0.23	0.04
Soil pH	−0.22	−0.11	−0.03	0.07	−0.36*	−0.60**	−0.63**	0.19
The soils at 0∼80 cm depth in the RS profile								
Clay	0.13	0.15	0.24	0.33	0.14	−0.07	−0.04	−0.21
Silt	0.30	0.41	0.22	0.26	0.26	0.53*	0.17	−0.32
Sand	−0.39	−0.51*	−0.38	−0.44	−0.35	−0.42	−0.12	0.44
Soil pH	−0.46	−0.44	−0.41	−0.31	−0.52*	0.05	0.42	0.41
The soils at below 80 cm depth in the RS profile								
Clay	−0.05	0.04	0.05	0.22	−0.16	0.18	−0.20	0.03
Silt	−0.11	−0.26	0.13	0.55**	−0.28	0.02	0.25	−0.04
Sand	0.07	0.15	−0.18	−0.62**	0.28	−0.11	−0.13	0.07
Soil pH	0.21	0.37*	−0.05	−0.77**	0.55**	−0.01	−0.44**	−0.05

**Notes.**

Different-sized particles include clay, silt, and sand sized particles. Oxides includes Al_2_O_3_, MgO, TiO_2_, Fe_2_O_3_, K_2_O, Na_2_O, CaO, and SiO_2_.

YS yellow soil LRS Lateritic red soil RS red soil

## Conclusions

The K factors in the red soils were significantly lower than those in the yellow soils and significantly higher than those in the lateritic red soils. This result showed that soil erodibility significantly decreased with the progress of soil evolution. Although the SOC concentrations between the three soils were not significantly different, the distribution of them was affected by soil erosion and soil physicochemical properties (depended on soil evolution). In the deep soils (below 80 cm depth), the SON concentrations in the yellow soils were twice higher than those in the red soils and lateritic red soils, which was mainly attributed to the re-assimilation of inorganic N through microbial immobilization. The concentrations of soil major elements in the three soil types were almost lower than the background values of major elements in the mean soils of Fujian Province. Moreover, the concentrations of most major elements in the yellows were significantly larger than those in the red soils and lateritic red soils. The variation of soil major elements in the surface layer of the yellow soils was affected by both soil erosion and soil evolution, while it in the deep soils was mainly affected by soil evolution. These results suggest that yellow soils have a higher level in soil nutrients and soil erodibility compared to the red soils and lateritic red soils under the erosion region of Southeast China.

##  Supplemental Information

10.7717/peerj.11630/supp-1 Supplemental Information 1 Raw data

10.7717/peerj.11630/supp-2 Supplemental Information 2 Soil properties of all samples in the three profiles The unit of the K factor is ***t acre h/100 acre/ft/tanf/in***. YS, yellow soil; LRS, lateritic red soil; RS, red soil.

## Additional Information and Declarations

### Data Availability

The following information was supplied regarding data availability: Raw measurements are available in the Supplemental Files.
