# Supplementary material for: Distribution of soil nutrients and erodibility factor under different soil types in an erosion region of Southeast China
Source: PeerJ. 2021 Jun 16;9:e11630. doi: 10.7717/peerj.11630 (PMC8214394; doi:10.7717/peerj.11630)
Supplement: Supplemental Information 2 — The unit of the K factor is t acre h/100 acre/ft/tanf/in. YS, yellow soil; LRS, lateritic red soil; RS, red soil. [file peerj-09-11630-s002.docx]

| Site | Depth (cm) | SON  (g/kg) | SOC  (g/kg) | C/N | δ^15^N  (‰) | Clay  (%) | Silt  (%) | Sand  (%) | Soil pH | K factor | AL_2_O_3_  (%) | MgO  (%) | TiO_2_  (%) | Fe_2_O_3_  (%) | K_2_O  (%) | Na_2_O  (%) | CaO  (%) | SIO_2_  (%) |
| --- | --- | --- | --- | --- | --- | --- | --- | --- | --- | --- | --- | --- | --- | --- | --- | --- | --- | --- |
| YS | -5 | 0.88 | 7.61 | 8.63 | 2.30 | 9.6 | 65.6 | 24.8 | 4.3 | 0.408 | 7.51 | 0.18 | 0.94 | 1.80 | 0.76 | 0.12 | 0.076 | 65.9 |
| YS | -10 | 0.66 | 5.67 | 8.54 | 0.79 | 11.6 | 63.0 | 25.4 | 4.4 | 0.407 | 7.15 | 0.17 | 0.91 | 1.69 | 0.73 | 0.12 | 0.074 | 73.6 |
| YS | -15 | 0.67 | 5.78 | 8.59 | 1.24 | 11.7 | 63.6 | 24.7 | 4.3 | 0.409 | 7.28 | 0.17 | 0.94 | 1.62 | 0.75 | 0.12 | 0.072 | 73.5 |
| YS | -20 | 0.65 | 5.89 | 9.06 | 2.65 | 11.4 | 62.5 | 26.1 | 4.4 | 0.405 | 7.07 | 0.16 | 0.90 | 1.67 | 0.73 | 0.12 | 0.072 | 73.7 |
| YS | -25 | 0.61 | 5.47 | 8.96 | 2.15 | 11.8 | 61.8 | 26.5 | 4.3 | 0.403 | 7.29 | 0.16 | 0.91 | 1.73 | 0.74 | 0.11 | 0.072 | 73.4 |
| YS | -30 | 0.47 | 3.82 | 8.10 | 3.46 | 11.0 | 63.7 | 25.3 | 4.2 | 0.414 | 6.76 | 0.15 | 0.85 | 2.16 | 0.66 | 0.11 | 0.064 | 73.9 |
| YS | -35 | 0.50 | 3.83 | 7.75 | 3.12 | 12.9 | 64.0 | 23.1 | 4.0 |  | 7.76 | 0.17 | 0.83 | 5.80 | 0.69 | 0.10 | 0.058 | 69.2 |
| YS | -40 | 0.54 | 4.02 | 7.41 | 4.52 | 13.6 | 68.5 | 18.0 | 3.9 |  | 9.98 | 0.21 | 0.92 | 6.78 | 0.78 | 0.12 | 0.062 | 65.7 |
| YS | -45 | 0.52 | 3.12 | 6.06 | 4.43 | 12.3 | 69.2 | 18.5 | 3.8 |  | 10.87 | 0.24 | 0.95 | 5.20 | 0.83 | 0.12 | 0.069 | 66.4 |
| YS | -50 | 0.47 | 2.49 | 5.26 | 4.76 | 13.9 | 65.0 | 21.1 | 3.8 |  | 10.44 | 0.23 | 0.91 | 4.37 | 0.78 | 0.11 | 0.065 | 67.8 |
| YS | -55 | 0.48 | 2.66 | 5.54 | 4.58 | 11.0 | 67.8 | 21.1 | 3.8 |  | 11.27 | 0.24 | 0.99 | 4.56 | 0.85 | 0.12 | 0.065 | 66.6 |
| YS | -60 | 0.46 | 2.44 | 5.27 | 5.39 | 10.6 | 70.1 | 19.3 | 3.8 |  | 10.94 | 0.24 | 0.96 | 4.21 | 0.83 | 0.11 | 0.065 | 67.4 |
| YS | -65 | 0.49 | 2.91 | 5.93 | 5.17 | 14.6 | 66.8 | 18.7 | 3.8 |  | 10.76 | 0.23 | 0.95 | 4.05 | 0.83 | 0.12 | 0.065 | 67.7 |
| YS | -70 | 0.50 | 3.32 | 6.61 | 5.67 | 12.1 | 68.6 | 19.3 | 3.8 |  | 11.45 | 0.24 | 1.00 | 3.88 | 0.89 | 0.11 | 0.064 | 67.0 |
| YS | -75 | 0.52 | 3.39 | 6.51 | 5.04 | 12.0 | 68.3 | 19.8 | 3.8 |  | 10.94 | 0.23 | 0.95 | 3.81 | 0.83 | 0.11 | 0.065 | 67.7 |
| YS | -80 | 0.51 | 3.03 | 5.88 | 4.68 | 13.4 | 67.3 | 19.3 | 3.8 |  | 11.04 | 0.24 | 0.92 | 3.65 | 0.82 | 0.11 | 0.068 | 67.8 |
| YS | -85 | 0.45 | 2.35 | 5.19 | 4.82 | 13.7 | 67.4 | 19.0 | 3.8 |  | 11.28 | 0.24 | 0.97 | 3.86 | 0.86 | 0.13 | 0.072 | 67.4 |
| YS | -90 | 0.49 | 2.96 | 6.04 | 5.13 | 14.4 | 67.0 | 18.6 | 3.8 |  | 11.09 | 0.23 | 0.94 | 3.70 | 0.85 | 0.11 | 0.059 | 67.7 |
| YS | -95 | 0.47 | 2.52 | 5.35 | 4.67 | 13.2 | 66.8 | 20.1 | 3.8 |  | 10.73 | 0.22 | 0.87 | 3.57 | 0.83 | 0.10 | 0.056 | 68.4 |
| YS | -100 | 0.49 | 2.64 | 5.43 | 5.15 | 11.6 | 68.7 | 19.7 | 3.8 |  | 10.84 | 0.22 | 0.88 | 3.47 | 0.84 | 0.10 | 0.055 | 68.3 |
| YS | -105 | 0.55 | 3.01 | 5.47 | 5.81 | 9.8 | 71.1 | 19.1 | 3.8 |  | 11.04 | 0.23 | 0.90 | 3.60 | 0.88 | 0.11 | 0.058 | 67.9 |
| YS | -110 | 0.47 | 2.42 | 5.12 | 4.54 | 10.1 | 70.8 | 19.1 | 3.8 |  | 11.36 | 0.23 | 0.89 | 3.58 | 0.88 | 0.11 | 0.057 | 67.7 |
| YS | -115 | 0.42 | 2.16 | 5.09 | 5.73 | 13.3 | 65.9 | 20.8 | 3.8 |  | 8.76 | 0.22 | 0.90 | 3.46 | 0.81 | 0.11 | 0.045 | 70.5 |
| YS | -120 | 0.43 | 2.15 | 5.01 | 5.05 | 10.6 | 69.6 | 19.8 | 3.8 |  | 10.61 | 0.21 | 0.87 | 3.41 | 0.83 | 0.11 | 0.055 | 68.7 |
| YS | -125 | 0.42 | 1.92 | 4.60 | 5.11 | 13.3 | 66.6 | 20.2 | 3.8 |  | 10.59 | 0.21 | 0.86 | 3.41 | 0.84 | 0.11 | 0.057 | 68.7 |
| YS | -130 | 0.44 | 2.07 | 4.77 | 5.20 | 13.3 | 68.0 | 18.7 | 3.8 |  | 10.77 | 0.22 | 0.93 | 3.45 | 0.86 | 0.10 | 0.056 | 68.4 |
| YS | -135 | 0.45 | 2.31 | 5.17 | 5.47 | 9.6 | 71.5 | 18.9 | 3.8 |  | 11.23 | 0.22 | 0.89 | 3.44 | 0.87 | 0.11 | 0.058 | 68.0 |
| YS | -140 | 0.41 | 2.07 | 5.08 | 4.85 | 11.1 | 72.1 | 16.8 | 3.8 |  | 11.21 | 0.22 | 0.89 | 3.41 | 0.88 | 0.11 | 0.059 | 68.0 |
| YS | -145 | 0.45 | 2.37 | 5.26 | 5.84 | 11.6 | 70.0 | 18.4 | 3.8 |  | 11.44 | 0.22 | 0.86 | 3.82 | 0.88 | 0.11 | 0.060 | 67.4 |
| YS | -150 | 0.45 | 2.32 | 5.21 | 4.45 | 11.4 | 68.3 | 20.3 | 3.8 |  | 10.35 | 0.22 | 0.90 | 3.43 | 0.88 | 0.10 | 0.058 | 68.8 |
| YS | -155 | 0.42 | 2.02 | 4.82 | 4.46 | 10.2 | 70.7 | 19.1 | 3.8 |  | 10.52 | 0.22 | 0.94 | 3.45 | 0.91 | 0.10 | 0.059 | 68.6 |
| YS | -160 | 0.43 | 2.13 | 4.92 | 5.15 | 13.0 | 70.0 | 17.0 | 3.8 |  | 11.37 | 0.22 | 0.89 | 3.45 | 0.90 | 0.10 | 0.058 | 67.8 |
| YS | -165 | 0.46 | 2.21 | 4.84 | 4.90 | 10.5 | 70.9 | 18.6 | 4.0 |  | 10.97 | 0.22 | 0.87 | 3.35 | 0.90 | 0.10 | 0.067 | 68.3 |
| YS | -170 | 0.46 | 2.17 | 4.66 | 5.00 | 14.2 | 69.1 | 16.7 | 3.9 |  | 10.54 | 0.23 | 0.92 | 3.48 | 0.94 | 0.11 | 0.058 | 68.5 |
| YS | -175 | 0.46 | 2.13 | 4.58 | 4.91 | 14.5 | 68.1 | 17.4 | 3.9 |  | 12.12 | 0.24 | 0.93 | 3.72 | 0.96 | 0.11 | 0.062 | 66.7 |
| YS | -180 | 0.45 | 2.15 | 4.74 | 5.22 | 14.3 | 69.1 | 16.6 | 4.0 |  | 11.33 | 0.22 | 0.87 | 3.47 | 0.92 | 0.11 | 0.070 | 67.8 |
| YS | -185 | 0.44 | 1.97 | 4.45 | 4.50 | 11.5 | 69.6 | 18.9 | 4.1 |  | 10.60 | 0.22 | 0.90 | 3.42 | 0.92 | 0.11 | 0.060 | 68.6 |
| YS | -190 | 0.47 | 2.17 | 4.60 | 4.12 | 14.4 | 66.9 | 18.7 | 4.1 |  | 11.32 | 0.23 | 0.95 | 3.58 | 0.98 | 0.11 | 0.068 | 67.5 |
| YS | -195 | 0.48 | 2.22 | 4.65 | 4.97 | 12.1 | 71.2 | 16.7 | 4.2 |  | 11.44 | 0.22 | 0.89 | 3.38 | 0.94 | 0.11 | 0.076 | 67.7 |
| YS | -200 | 0.43 | 1.84 | 4.25 | 5.23 | 12.6 | 71.1 | 16.3 | 4.0 |  | 11.61 | 0.22 | 0.87 | 3.38 | 0.93 | 0.11 | 0.067 | 67.6 |
| YS | -205 | 0.49 | 2.24 | 4.60 | 4.80 | 17.4 | 66.7 | 15.9 | 4.3 |  | 12.19 | 0.24 | 0.94 | 3.52 | 1.00 | 0.12 | 0.087 | 66.7 |
| YS | -210 | 0.45 | 2.05 | 4.52 | 5.08 | 12.6 | 69.6 | 17.8 | 4.3 |  | 11.23 | 0.23 | 0.94 | 3.41 | 0.99 | 0.12 | 0.083 | 67.8 |
| YS | -215 | 0.44 | 2.10 | 4.79 | 4.41 | 14.9 | 67.7 | 17.4 | 4.4 |  | 11.55 | 0.23 | 0.92 | 3.48 | 0.99 | 0.12 | 0.085 | 67.4 |
| YS | -220 | 0.52 | 2.94 | 5.61 | 5.36 | 11.0 | 71.5 | 17.5 | 4.2 |  | 11.92 | 0.23 | 0.88 | 3.40 | 0.97 | 0.11 | 0.082 | 67.1 |
| YS | -225 | 0.40 | 1.67 | 4.17 | 4.61 | 13.0 | 71.0 | 15.9 | 4.3 |  | 11.24 | 0.23 | 0.95 | 3.30 | 0.95 | 0.12 | 0.075 | 68.0 |
| YS | -230 | 0.41 | 1.88 | 4.55 | 4.96 | 13.3 | 70.2 | 16.5 | 4.4 |  | 11.00 | 0.22 | 0.90 | 3.17 | 0.94 | 0.11 | 0.076 | 68.4 |
| YS | -235 | 0.43 | 2.07 | 4.78 | 4.90 | 12.6 | 69.9 | 17.5 | 4.4 |  | 11.67 | 0.23 | 0.91 | 3.28 | 0.97 | 0.11 | 0.086 | 67.5 |
| YS | -240 | 0.43 | 2.16 | 5.09 | 4.51 | 13.1 | 70.7 | 16.1 | 4.5 |  | 10.96 | 0.22 | 0.88 | 3.31 | 0.95 | 0.11 | 0.077 | 68.3 |
| YS | -245 | 0.41 | 1.73 | 4.16 | 4.56 | 12.4 | 68.5 | 19.1 | 4.5 |  | 10.77 | 0.22 | 0.88 | 3.15 | 0.93 | 0.11 | 0.077 | 68.7 |
| YS | -250 | 0.44 | 2.02 | 4.61 | 4.82 | 14.0 | 70.5 | 15.6 | 4.6 |  | 12.38 | 0.23 | 0.94 | 3.26 | 0.98 | 0.11 | 0.080 | 66.8 |
| YS | -255 | 0.43 | 1.93 | 4.48 | 4.52 | 13.5 | 68.6 | 17.9 | 4.6 |  | 11.10 | 0.22 | 0.91 | 3.24 | 0.96 | 0.11 | 0.080 | 68.2 |
| YS | -260 | 0.44 | 1.97 | 4.52 | 4.78 | 13.5 | 68.6 | 17.9 | 4.5 |  | 11.38 | 0.22 | 0.95 | 3.30 | 0.98 | 0.12 | 0.082 | 67.8 |
| YS | -265 | 0.43 | 1.97 | 4.60 | 4.99 | 14.1 | 67.9 | 18.0 | 4.6 |  | 11.04 | 0.22 | 0.94 | 3.21 | 0.97 | 0.11 | 0.080 | 68.2 |
| YS | -270 | 0.43 | 1.83 | 4.30 | 4.53 | 14.0 | 69.1 | 16.9 | 4.6 |  | 11.54 | 0.21 | 0.95 | 3.24 | 1.01 | 0.13 | 0.076 | 67.7 |
| YS | -275 | 0.50 | 2.90 | 5.79 | 5.00 | 13.7 | 68.3 | 18.0 | 4.6 |  | 11.08 | 0.22 | 0.93 | 3.16 | 0.96 | 0.11 | 0.079 | 68.2 |
| YS | -280 | 0.43 | 1.89 | 4.37 | 4.62 | 14.9 | 67.9 | 17.2 | 4.6 |  | 11.32 | 0.22 | 0.96 | 3.23 | 0.98 | 0.11 | 0.081 | 67.9 |
| YS | -285 | 0.42 | 2.20 | 5.21 | 4.68 | 12.6 | 66.4 | 21.1 | 4.6 |  | 10.65 | 0.21 | 0.93 | 3.10 | 0.94 | 0.11 | 0.081 | 68.8 |
| YS | -290 | 0.43 | 1.88 | 4.37 | 4.90 | 13.7 | 67.9 | 18.4 | 4.6 |  | 8.13 | 0.21 | 0.96 | 3.19 | 0.98 | 0.13 | 0.043 | 71.2 |
| YS | -295 | 0.38 | 1.49 | 3.96 | 4.43 | 12.1 | 67.8 | 20.1 | 4.7 |  | 11.03 | 0.21 | 0.86 | 3.08 | 0.96 | 0.11 | 0.078 | 68.5 |
| YS | -300 | 0.42 | 2.14 | 5.07 | 5.27 | 12.1 | 68.4 | 19.6 | 4.7 |  | 11.17 | 0.19 | 0.93 | 3.41 | 0.99 | 0.14 | 0.068 | 67.9 |
| LRS | -5 | 0.74 | 9.62 | 12.93 | 3.69 | 15.3 | 59.1 | 25.6 | 4.9 | 0.372 | 7.30 | 0.085 | 0.25 | 1.34 | 0.50 | 0.050 | 0.039 | 74.5 |
| LRS | -10 | 0.79 | 10.68 | 13.46 | 2.89 | 12.6 | 58.7 | 28.7 | 4.8 | 0.360 | 5.87 | 0.081 | 0.24 | 1.27 | 0.47 | 0.065 | 0.031 | 75.9 |
| LRS | -15 | 0.76 | 9.99 | 13.13 | 3.18 | 14.0 | 55.5 | 30.4 | 4.8 | 0.355 | 7.59 | 0.098 | 0.31 | 1.54 | 0.59 | 0.056 | 0.030 | 73.8 |
| LRS | -20 | 0.47 | 5.72 | 12.18 | 3.48 | 13.9 | 54.6 | 31.4 | 4.7 | 0.375 | 7.70 | 0.078 | 0.22 | 1.26 | 0.45 | 0.048 | 0.026 | 74.6 |
| LRS | -25 | 0.50 | 6.72 | 13.42 | 3.21 | 13.7 | 55.4 | 31.0 | 4.7 | 0.374 | 8.11 | 0.073 | 0.25 | 1.41 | 0.50 | 0.046 | 0.023 | 73.9 |
| LRS | -30 | 0.28 | 3.51 | 12.40 | 3.67 | 15.3 | 54.2 | 30.5 | 4.7 | 0.378 | 6.32 | 0.067 | 0.17 | 1.04 | 0.47 | 0.042 | 0.017 | 76.5 |
| LRS | -35 | 0.32 | 4.01 | 12.63 | 5.30 | 14.8 | 64.5 | 20.7 | 4.7 |  | 7.93 | 0.076 | 0.22 | 1.23 | 0.48 | 0.045 | 0.017 | 74.6 |
| LRS | -40 | 0.32 | 3.75 | 11.70 | 4.36 | 15.6 | 66.1 | 18.3 | 4.6 |  | 10.23 | 0.102 | 0.29 | 1.58 | 0.62 | 0.049 | 0.018 | 71.7 |
| LRS | -45 | 0.24 | 2.63 | 10.78 | 4.59 | 12.8 | 62.2 | 25.0 | 4.6 |  | 7.74 | 0.074 | 0.20 | 1.14 | 0.42 | 0.040 | 0.015 | 75.1 |
| LRS | -50 | 0.16 | 2.11 | 13.44 | 4.20 | 10.9 | 66.0 | 23.1 | 4.6 |  | 4.48 | 0.054 | 0.20 | 1.27 | 0.42 | 0.046 | 0.007 | 78.3 |
| LRS | -55 | 0.20 | 2.62 | 12.79 | 5.55 | 15.7 | 66.1 | 18.3 | 4.5 |  | 8.41 | 0.055 | 0.23 | 1.49 | 0.46 | 0.048 | 0.012 | 74.0 |
| LRS | -60 | 0.16 | 1.85 | 11.56 | 4.42 | 12.1 | 61.7 | 26.2 | 4.5 |  | 6.58 | 0.064 | 0.14 | 1.02 | 0.36 | 0.033 | 0.012 | 76.6 |
| LRS | -65 | 0.17 | 2.03 | 11.96 | 3.11 | 11.8 | 59.2 | 28.9 | 4.5 |  | 8.25 | 0.080 | 0.18 | 1.33 | 0.38 | 0.039 | 0.017 | 74.5 |
| LRS | -70 | 0.11 | 1.28 | 11.23 | 4.42 | 12.6 | 66.2 | 21.2 | 4.4 |  | 6.93 | 0.089 | 0.16 | 1.28 | 0.35 | 0.162 | 0.058 | 75.8 |
| LRS | -75 | 0.17 | 1.68 | 10.02 | 3.17 | 13.3 | 72.0 | 14.7 | 4.4 |  | 11.84 | 0.129 | 0.22 | 1.87 | 0.52 | 0.134 | 0.043 | 70.1 |
| LRS | -80 | 0.17 | 1.75 | 10.27 | 4.47 | 15.8 | 71.5 | 12.7 | 4.4 |  | 12.19 | 0.141 | 0.24 | 2.09 | 0.55 | 0.169 | 0.058 | 69.4 |
| LRS | -85 | 0.14 | 1.32 | 9.71 | 2.44 | 15.1 | 67.7 | 17.2 | 4.4 |  | 9.76 | 0.112 | 0.18 | 1.65 | 0.41 | 0.133 | 0.045 | 72.6 |
| LRS | -90 | 0.15 | 1.74 | 11.40 | 2.10 | 13.9 | 68.1 | 18.0 | 4.3 |  | 12.16 | 0.128 | 0.23 | 2.06 | 0.53 | 0.180 | 0.067 | 69.5 |
| LRS | -95 | 0.18 | 1.83 | 10.35 | 1.18 | 11.8 | 59.3 | 29.0 | 4.5 |  | 12.07 | 0.141 | 0.21 | 1.97 | 0.73 | 0.158 | 0.054 | 69.5 |
| LRS | -100 | 0.23 | 2.49 | 11.01 | 1.44 | 12.5 | 62.6 | 24.9 | 4.5 |  | 8.72 | 0.092 | 0.15 | 1.63 | 0.64 | 0.042 | 0.010 | 73.5 |
| LRS | -105 | 0.12 | 1.25 | 10.21 | 1.52 | 15.4 | 68.3 | 16.4 | 4.7 |  | 9.52 | 0.100 | 0.19 | 1.84 | 0.48 | 0.045 | 0.011 | 72.7 |
| LRS | -110 | 0.20 | 1.78 | 9.08 | 2.12 | 17.6 | 70.5 | 11.8 | 4.9 |  | 10.61 | 0.114 | 0.21 | 2.04 | 0.50 | 0.050 | 0.015 | 71.1 |
| LRS | -115 | 0.14 | 1.80 | 12.53 | 2.87 | 20.3 | 64.4 | 15.3 | 5.0 |  | 9.69 | 0.116 | 0.22 | 2.15 | 0.51 | 0.041 | 0.009 | 72.1 |
| LRS | -120 | 0.15 | 1.34 | 9.20 | 3.18 | 19.2 | 62.5 | 18.2 | 5.1 |  | 5.51 | 0.083 | 0.17 | 1.74 | 0.40 | 0.039 | 0.007 | 76.9 |
| LRS | -125 | 0.20 | 2.81 | 14.08 | 4.50 | 19.5 | 64.7 | 15.7 | 4.8 |  | 15.81 | 0.102 | 0.24 | 2.69 | 0.60 | 0.066 | 0.015 | 65.2 |
| LRS | -130 | 0.25 | 3.04 | 12.32 | 4.81 | 17.6 | 77.3 | 5.2 | 4.8 |  | 15.98 | 0.173 | 0.27 | 3.26 | 0.75 | 0.062 | 0.011 | 64.2 |
| LRS | -135 | 0.21 | 2.22 | 10.82 | 3.69 | 18.6 | 71.1 | 10.4 | 4.8 |  | 13.67 | 0.174 | 0.27 | 3.21 | 0.76 | 0.053 | 0.010 | 66.6 |
| LRS | -140 | 0.19 | 1.85 | 9.90 | 2.87 | 19.9 | 65.2 | 14.9 | 4.8 |  | 14.16 | 0.136 | 0.21 | 2.65 | 0.58 | 0.050 | 0.010 | 67.0 |
| LRS | -145 | 0.22 | 2.37 | 10.79 | 2.33 | 21.7 | 67.5 | 10.8 | 4.8 |  | 13.39 | 0.106 | 0.17 | 2.21 | 0.46 | 0.040 | 0.012 | 68.4 |
| LRS | -150 | 0.22 | 2.26 | 10.48 | 3.63 | 17.7 | 72.1 | 10.2 | 4.7 |  | 14.30 | 0.147 | 0.21 | 3.04 | 0.62 | 0.050 | 0.010 | 66.4 |
| LRS | -155 | 0.24 | 2.28 | 9.32 | 2.38 | 17.8 | 70.3 | 12.0 | 4.8 |  | 19.40 | 0.208 | 0.30 | 3.94 | 1.02 | 0.062 | 0.010 | 59.8 |
| LRS | -160 | 0.18 | 1.88 | 10.64 | 2.28 | 14.8 | 65.1 | 20.1 | 4.8 |  | 11.95 | 0.122 | 0.17 | 2.39 | 0.62 | 0.050 | 0.012 | 69.5 |
| LRS | -165 | 0.19 | 2.04 | 10.87 | 3.26 | 18.1 | 59.9 | 22.1 | 4.8 |  | 15.52 | 0.143 | 0.19 | 2.82 | 0.68 | 0.056 | 0.010 | 65.4 |
| LRS | -170 | 0.17 | 1.81 | 10.93 | 2.92 | 19.2 | 67.4 | 13.4 | 4.8 |  | 10.94 | 0.105 | 0.14 | 2.27 | 0.44 | 0.042 | 0.008 | 70.9 |
| LRS | -175 | 0.12 | 1.77 | 14.93 | 2.93 | 18.2 | 68.5 | 13.3 | 4.8 |  | 11.99 | 0.111 | 0.16 | 2.40 | 0.48 | 0.056 | 0.011 | 69.6 |
| LRS | -180 | 0.15 | 1.86 | 11.98 | 3.54 | 11.7 | 61.8 | 26.4 | 4.9 |  | 12.77 | 0.119 | 0.18 | 2.80 | 0.53 | 0.045 | 0.008 | 68.4 |
| LRS | -185 | 0.12 | 1.34 | 11.48 | 4.03 | 12.6 | 58.2 | 29.2 | 4.8 |  | 11.26 | 0.129 | 0.16 | 2.18 | 1.22 | 0.072 | 0.011 | 69.8 |
| LRS | -190 | 0.21 | 2.40 | 11.36 | 4.49 | 17.4 | 61.7 | 20.9 | 4.7 |  | 13.74 | 0.119 | 0.16 | 2.10 | 1.05 | 0.065 | 0.011 | 67.5 |
| LRS | -195 | 0.15 | 1.97 | 13.07 | 5.41 | 14.7 | 61.8 | 23.6 | 4.8 |  | 10.76 | 0.037 | 0.14 | 2.01 | 0.61 | 0.071 | 0.006 | 71.2 |
| LRS | -200 | 0.26 | 2.57 | 9.79 | 5.13 | 19.8 | 59.7 | 20.5 | 4.8 |  | 13.20 | 0.113 | 0.15 | 1.81 | 0.60 | 0.138 | 0.045 | 68.7 |
| LRS | -205 | 0.22 | 2.33 | 10.79 | 5.73 | 20.9 | 65.9 | 13.3 | 4.7 |  | 16.70 | 0.127 | 0.27 | 3.40 | 1.18 | 0.181 | 0.030 | 62.6 |
| LRS | -210 | 0.24 | 2.93 | 12.10 | 4.12 | 18.1 | 66.2 | 15.7 | 4.7 |  | 14.91 | 0.111 | 0.19 | 2.38 | 1.15 | 0.177 | 0.045 | 65.7 |
| LRS | -215 | 0.20 | 2.35 | 11.55 | 4.32 | 17.0 | 63.9 | 19.1 | 4.7 |  | 17.15 | 0.146 | 0.20 | 2.56 | 1.14 | 0.172 | 0.049 | 63.4 |
| LRS | -220 | 0.15 | 2.02 | 13.45 | 2.57 | 16.3 | 70.5 | 13.2 | 4.7 |  | 13.08 | 0.113 | 0.14 | 2.02 | 0.60 | 0.139 | 0.043 | 68.7 |
| LRS | -225 | 0.19 | 2.32 | 12.43 | 1.62 | 11.0 | 59.4 | 29.6 | 4.7 |  | 16.81 | 0.130 | 0.22 | 3.14 | 1.03 | 0.167 | 0.028 | 63.2 |
| LRS | -230 | 0.19 | 2.20 | 11.38 | -1.25 | 12.8 | 69.4 | 17.8 | 4.7 |  | 16.44 | 0.118 | 0.17 | 2.66 | 1.08 | 0.157 | 0.024 | 64.1 |
| LRS | -235 | 0.14 | 1.79 | 13.22 | -0.73 | 10.3 | 68.6 | 21.0 | 4.7 |  | 16.07 | 0.092 | 0.19 | 2.91 | 1.34 | 0.177 | 0.025 | 64.0 |
| LRS | -240 | 0.13 | 1.59 | 12.57 | 0.03 | 14.0 | 63.1 | 22.9 | 4.7 |  | 13.38 | 0.047 | 0.17 | 2.68 | 0.91 | 0.150 | 0.022 | 67.5 |
| LRS | -245 | 0.11 | 1.42 | 12.44 | 1.51 | 14.0 | 68.9 | 17.1 | 4.7 |  | 16.63 | 0.132 | 0.21 | 3.20 | 1.12 | 0.174 | 0.034 | 63.4 |
| LRS | -250 | 0.12 | 1.36 | 11.55 | 1.24 | 13.8 | 58.2 | 28.1 | 4.7 |  | 15.85 | 0.135 | 0.21 | 2.41 | 1.05 | 0.175 | 0.050 | 65.0 |
| LRS | -255 | 0.08 | 0.98 | 11.55 | -0.28 | 14.6 | 58.4 | 27.0 | 4.7 |  | 13.81 | 0.116 | 0.18 | 1.85 | 0.78 | 0.147 | 0.047 | 68.0 |
| LRS | -260 | 0.14 | 1.97 | 14.01 | 0.16 | 14.9 | 66.0 | 19.2 | 4.7 |  | 19.51 | 0.119 | 0.24 | 3.37 | 1.27 | 0.187 | 0.049 | 60.1 |
| LRS | -265 | 0.11 | 1.43 | 13.33 | 0.25 | 11.8 | 62.2 | 26.0 | 4.8 |  | 15.87 | 0.128 | 0.18 | 2.43 | 0.98 | 0.157 | 0.044 | 65.1 |
| LRS | -270 | 0.14 | 2.14 | 14.81 | 2.51 | 14.2 | 64.3 | 21.6 | 4.7 |  | 15.85 | 0.123 | 0.21 | 3.07 | 0.96 | 0.174 | 0.047 | 64.4 |
| LRS | -275 | 0.14 | 1.84 | 13.30 | 2.92 | 19.2 | 61.4 | 19.5 | 4.7 |  | 14.41 | 0.113 | 0.21 | 2.52 | 0.71 | 0.134 | 0.036 | 66.7 |
| LRS | -280 | 0.13 | 1.70 | 12.69 | 2.87 | 18.3 | 67.0 | 14.7 | 4.7 |  | 14.08 | 0.136 | 0.16 | 2.02 | 0.70 | 0.128 | 0.039 | 67.6 |
| LRS | -285 | 0.11 | 1.72 | 15.40 | 1.65 | 16.3 | 66.3 | 17.4 | 4.7 |  | 18.11 | 0.188 | 0.21 | 2.81 | 1.10 | 0.164 | 0.051 | 62.2 |
| LRS | -290 | 0.14 | 1.71 | 12.06 | 0.30 | 18.5 | 66.1 | 15.5 | 4.7 |  | 15.62 | 0.174 | 0.17 | 2.31 | 0.89 | 0.143 | 0.047 | 65.5 |
| LRS | -295 | 0.17 | 1.93 | 11.65 | 0.16 | 16.1 | 72.1 | 11.8 | 4.7 |  | 14.93 | 0.169 | 0.17 | 2.88 | 0.90 | 0.126 | 0.023 | 65.6 |
| LRS | -300 | 0.15 | 2.22 | 14.67 | 0.53 | 15.9 | 66.9 | 17.2 | 4.7 |  | 14.02 | 0.182 | 0.16 | 2.41 | 0.98 | 0.056 | 0.010 | 67.0 |
| RS | -5 | 0.91 | 14.42 | 15.76 | -0.80 | 12.1 | 65.3 | 22.6 | 4.2 | 0.348 | 14.04 | 0.17 | 0.80 | 3.83 | 0.46 | 0.078 | 0.041 | 64.1 |
| RS | -10 | 0.59 | 8.48 | 14.43 | 2.09 | 12.4 | 66.0 | 21.6 | 4.2 | 0.405 | 13.93 | 0.19 | 0.79 | 3.59 | 0.44 | 0.068 | 0.028 | 65.1 |
| RS | -15 | 0.58 | 9.73 | 16.90 | 2.37 | 11.3 | 63.7 | 25.0 | 4.2 | 0.386 | 9.67 | 0.15 | 0.64 | 2.77 | 0.38 | 0.065 | 0.023 | 70.3 |
| RS | -20 | 0.44 | 6.31 | 14.25 | 3.20 | 11.3 | 62.8 | 25.9 | 4.3 | 0.404 | 10.94 | 0.17 | 0.70 | 3.17 | 0.41 | 0.080 | 0.031 | 68.9 |
| RS | -25 | 0.52 | 8.39 | 16.09 | 2.95 | 11.1 | 67.3 | 21.6 | 4.2 | 0.409 | 11.42 | 0.17 | 0.63 | 2.91 | 0.39 | 0.104 | 0.056 | 68.5 |
| RS | -30 | 0.51 | 9.64 | 18.91 | 4.15 | 12.5 | 68.9 | 18.7 | 4.2 | 0.405 | 12.13 | 0.18 | 0.73 | 3.55 | 0.40 | 0.076 | 0.040 | 66.9 |
| RS | -35 | 0.68 | 11.46 | 16.95 | 4.81 | 13.4 | 64.9 | 21.7 | 4.3 |  | 8.46 | 0.14 | 0.55 | 2.56 | 0.31 | 0.073 | 0.048 | 71.7 |
| RS | -40 | 0.55 | 9.57 | 17.48 | 4.54 | 9.0 | 65.2 | 25.8 | 4.2 |  | 7.40 | 0.12 | 0.45 | 2.14 | 0.27 | 0.066 | 0.060 | 73.5 |
| RS | -45 | 0.54 | 9.99 | 18.66 | 4.04 | 14.3 | 64.1 | 21.6 | 4.2 |  | 11.14 | 0.15 | 0.68 | 3.17 | 0.40 | 0.067 | 0.032 | 68.4 |
| RS | -50 | 0.53 | 9.37 | 17.78 | 3.67 | 12.7 | 61.8 | 25.5 | 4.2 |  | 9.67 | 0.14 | 0.62 | 2.87 | 0.36 | 0.064 | 0.026 | 70.3 |
| RS | -55 | 0.52 | 8.99 | 17.38 | 4.62 | 13.0 | 63.6 | 23.4 | 4.2 |  | 12.36 | 0.17 | 0.74 | 3.56 | 0.42 | 0.068 | 0.028 | 66.8 |
| RS | -60 | 0.41 | 6.42 | 15.52 | 4.72 | 12.2 | 65.0 | 22.8 | 4.2 |  | 10.41 | 0.13 | 0.61 | 2.87 | 0.36 | 0.072 | 0.019 | 69.9 |
| RS | -65 | 0.46 | 7.33 | 15.79 | 4.77 | 10.0 | 64.1 | 25.9 | 4.2 |  | 10.86 | 0.14 | 0.61 | 2.72 | 0.38 | 0.058 | 0.022 | 69.5 |
| RS | -70 | 0.32 | 3.76 | 11.92 | 3.94 | 11.3 | 64.4 | 24.3 | 4.1 |  | 14.17 | 0.19 | 0.78 | 3.48 | 0.55 | 0.075 | 0.024 | 65.4 |
| RS | -75 | 0.25 | 2.85 | 11.28 | 3.09 | 11.7 | 65.9 | 22.4 | 4.2 |  | 10.76 | 0.17 | 0.68 | 3.05 | 0.52 | 0.088 | 0.018 | 69.4 |
| RS | -80 | 0.24 | 2.22 | 9.22 | 2.90 | 13.0 | 65.7 | 21.3 | 4.1 |  | 12.03 | 0.19 | 0.78 | 3.28 | 0.58 | 0.074 | 0.019 | 67.8 |
| RS | -85 | 0.20 | 2.15 | 10.51 | 2.89 | 12.6 | 65.1 | 22.3 | 4.1 |  | 11.58 | 0.16 | 0.62 | 2.81 | 0.50 | 0.066 | 0.024 | 69.0 |
| RS | -90 | 0.20 | 1.77 | 9.07 | 2.43 | 12.0 | 66.0 | 22.1 | 4.2 |  | 10.33 | 0.15 | 0.57 | 2.48 | 0.47 | 0.087 | 0.029 | 70.7 |
| RS | -95 | 0.25 | 2.38 | 9.54 | 3.87 | 12.1 | 64.7 | 23.2 | 4.2 |  | 11.43 | 0.17 | 0.62 | 2.68 | 0.53 | 0.062 | 0.031 | 69.2 |
| RS | -100 | 0.22 | 1.84 | 8.42 | 3.48 | 11.8 | 63.9 | 24.4 | 4.3 |  | 9.10 | 0.12 | 0.59 | 2.48 | 0.50 | 0.143 | 0.025 | 71.9 |
| RS | -105 | 0.25 | 2.43 | 9.89 | 3.14 | 11.9 | 64.8 | 23.3 | 4.2 |  | 10.83 | 0.15 | 0.63 | 2.76 | 0.55 | 0.071 | 0.027 | 69.7 |
| RS | -110 | 0.22 | 1.98 | 9.21 | 3.66 | 12.2 | 66.4 | 21.4 | 4.3 |  | 11.92 | 0.20 | 0.75 | 3.27 | 0.66 | 0.078 | 0.029 | 67.9 |
| RS | -115 | 0.20 | 1.89 | 9.25 | 3.28 | 9.2 | 67.4 | 23.4 | 4.2 |  | 12.96 | 0.15 | 0.68 | 2.90 | 0.60 | 0.067 | 0.036 | 67.4 |
| RS | -120 | 0.23 | 2.49 | 10.67 | 4.25 | 10.4 | 66.7 | 23.0 | 4.2 |  | 12.73 | 0.17 | 0.65 | 2.86 | 0.58 | 0.062 | 0.036 | 67.7 |
| RS | -125 | 0.16 | 1.39 | 8.90 | 4.10 | 8.0 | 63.2 | 28.8 | 4.3 |  | 8.85 | 0.11 | 0.41 | 1.97 | 0.41 | 0.069 | 0.033 | 73.0 |
| RS | -130 | 0.15 | 1.64 | 10.79 | 4.66 | 6.6 | 64.2 | 29.2 | 4.4 |  | 6.07 | 0.09 | 0.25 | 1.26 | 0.35 | 0.045 | 0.023 | 76.8 |
| RS | -135 | 0.19 | 1.77 | 9.52 | 3.33 | 10.5 | 64.4 | 25.2 | 4.3 |  | 9.62 | 0.15 | 0.48 | 2.33 | 0.59 | 0.063 | 0.028 | 71.6 |
| RS | -140 | 0.19 | 1.61 | 8.44 | 2.85 | 10.2 | 64.4 | 25.4 | 4.3 |  | 13.65 | 0.19 | 0.61 | 2.80 | 0.78 | 0.073 | 0.038 | 66.7 |
| RS | -145 | 0.21 | 2.54 | 12.01 | 2.48 | 10.1 | 62.9 | 26.9 | 4.3 |  | 12.29 | 0.19 | 0.60 | 2.45 | 0.74 | 0.066 | 0.033 | 68.4 |
| RS | -150 | 0.20 | 1.53 | 7.56 | 2.33 | 12.5 | 63.7 | 23.8 | 4.4 |  | 14.24 | 0.23 | 0.71 | 2.74 | 0.96 | 0.076 | 0.031 | 65.9 |
| RS | -155 | 0.20 | 1.57 | 7.81 | 3.26 | 11.2 | 65.5 | 23.3 | 4.3 |  | 11.50 | 0.18 | 0.57 | 2.05 | 0.78 | 0.058 | 0.022 | 69.7 |
| RS | -160 | 0.20 | 1.33 | 6.81 | 1.96 | 12.1 | 66.6 | 21.3 | 4.4 |  | 11.04 | 0.17 | 0.55 | 1.88 | 0.76 | 0.057 | 0.021 | 70.4 |
| RS | -165 | 0.27 | 1.72 | 6.33 | 2.78 | 12.4 | 66.1 | 21.5 | 4.2 |  | 13.47 | 0.22 | 0.69 | 2.25 | 0.97 | 0.074 | 0.021 | 67.1 |
| RS | -170 | 0.23 | 1.72 | 7.34 | 2.16 | 13.5 | 66.7 | 19.8 | 4.2 |  | 11.36 | 0.20 | 0.60 | 1.92 | 0.88 | 0.063 | 0.018 | 69.8 |
| RS | -175 | 0.27 | 2.33 | 8.63 | 2.30 | 11.3 | 64.5 | 24.1 | 4.3 |  | 12.34 | 0.19 | 0.58 | 1.83 | 0.83 | 0.066 | 0.021 | 68.9 |
| RS | -180 | 0.17 | 1.79 | 10.37 | 2.60 | 10.3 | 66.0 | 23.7 | 4.2 |  | 7.87 | 0.14 | 0.66 | 2.10 | 0.97 | 0.074 | 0.013 | 73.0 |
| RS | -185 | 0.22 | 1.49 | 6.87 | 2.03 | 9.8 | 67.4 | 22.9 | 4.2 |  | 13.09 | 0.23 | 0.70 | 2.24 | 1.01 | 0.078 | 0.022 | 67.5 |
| RS | -190 | 0.20 | 1.52 | 7.49 | 3.12 | 13.0 | 63.4 | 23.6 | 4.3 |  | 13.01 | 0.22 | 0.68 | 2.12 | 0.98 | 0.076 | 0.020 | 67.7 |
| RS | -195 | 0.23 | 2.03 | 8.86 | 1.94 | 10.7 | 62.5 | 26.8 | 4.4 |  | 12.89 | 0.22 | 0.68 | 2.11 | 0.98 | 0.082 | 0.023 | 67.8 |
| RS | -200 | 0.25 | 1.89 | 7.54 | 1.89 | 11.1 | 65.0 | 23.9 | 4.4 |  | 12.54 | 0.18 | 0.63 | 2.02 | 0.92 | 0.090 | 0.021 | 68.4 |
| RS | -205 | 0.24 | 2.21 | 9.18 | 2.92 | 11.1 | 63.2 | 25.7 | 4.4 |  | 12.93 | 0.23 | 0.71 | 2.20 | 1.00 | 0.075 | 0.022 | 67.6 |
| RS | -210 | 0.26 | 3.10 | 11.91 | 3.61 | 10.1 | 62.5 | 27.5 | 4.5 |  | 11.84 | 0.19 | 0.55 | 1.79 | 0.83 | 0.062 | 0.019 | 69.4 |
| RS | -215 | 0.20 | 1.85 | 9.19 | 3.92 | 10.9 | 60.9 | 28.1 | 4.6 |  | 10.03 | 0.16 | 0.56 | 1.74 | 0.82 | 0.080 | 0.015 | 71.4 |
| RS | -220 | 0.25 | 2.67 | 10.68 | 3.92 | 10.1 | 62.5 | 27.4 | 4.5 |  | 12.50 | 0.22 | 0.66 | 1.95 | 1.00 | 0.070 | 0.022 | 68.3 |
| RS | -225 | 0.25 | 1.64 | 6.60 | 3.52 | 11.4 | 62.3 | 26.3 | 4.4 |  | 13.18 | 0.22 | 0.67 | 1.91 | 0.98 | 0.072 | 0.023 | 67.8 |
| RS | -230 | 0.31 | 2.44 | 7.83 | 3.68 | 10.4 | 61.2 | 28.4 | 4.5 |  | 12.87 | 0.22 | 0.68 | 1.76 | 1.00 | 0.069 | 0.022 | 68.1 |
| RS | -235 | 0.26 | 1.55 | 5.86 | 2.79 | 9.5 | 61.8 | 28.7 | 4.4 |  | 13.03 | 0.23 | 0.64 | 1.74 | 1.04 | 0.071 | 0.024 | 68.1 |
| RS | -240 | 0.24 | 1.66 | 6.93 | 2.16 | 10.4 | 64.7 | 24.9 | 4.4 |  | 12.45 | 0.22 | 0.68 | 1.81 | 1.10 | 0.082 | 0.023 | 68.5 |
| RS | -245 | 0.23 | 2.04 | 8.70 | 4.94 | 9.7 | 65.6 | 24.7 | 4.5 |  | 13.86 | 0.19 | 0.66 | 1.84 | 1.03 | 0.080 | 0.024 | 67.1 |
| RS | -250 | 0.21 | 1.02 | 4.86 | 1.70 | 10.4 | 65.6 | 24.0 | 4.4 |  | 13.68 | 0.25 | 0.72 | 1.94 | 1.14 | 0.074 | 0.023 | 67.1 |
| RS | -255 | 0.23 | 1.87 | 8.29 | 1.88 | 10.8 | 63.1 | 26.1 | 4.5 |  | 12.70 | 0.21 | 0.60 | 1.55 | 1.01 | 0.066 | 0.020 | 68.6 |
| RS | -260 | 0.24 | 1.41 | 5.91 | 0.29 | 10.5 | 64.4 | 25.1 | 4.5 |  | 11.11 | 0.20 | 0.60 | 1.56 | 1.02 | 0.061 | 0.018 | 70.3 |
| RS | -265 | 0.23 | 1.65 | 7.07 | 0.88 | 9.8 | 65.1 | 25.2 | 4.4 |  | 14.69 | 0.26 | 0.72 | 1.82 | 1.19 | 0.071 | 0.023 | 66.1 |
| RS | -270 | 0.19 | 1.46 | 7.50 | 2.83 | 10.8 | 62.7 | 26.5 | 4.4 |  | 14.20 | 0.25 | 0.71 | 1.94 | 1.18 | 0.072 | 0.022 | 66.5 |
| RS | -275 | 0.22 | 1.86 | 8.39 | 3.44 | 11.5 | 64.6 | 23.9 | 4.4 |  | 11.25 | 0.20 | 0.57 | 1.45 | 0.95 | 0.068 | 0.022 | 70.3 |
| RS | -280 | 0.28 | 2.76 | 9.84 | 3.51 | 11.5 | 60.7 | 27.8 | 4.4 |  | 13.20 | 0.22 | 0.67 | 1.62 | 1.03 | 0.070 | 0.021 | 67.9 |
| RS | -285 | 0.19 | 1.67 | 8.72 | 3.21 | 13.1 | 61.6 | 25.3 | 4.5 |  | 13.69 | 0.23 | 0.66 | 1.66 | 1.09 | 0.083 | 0.021 | 67.4 |
| RS | -290 | 0.24 | 1.93 | 8.04 | 1.76 | 11.2 | 60.2 | 28.5 | 4.5 |  | 11.56 | 0.18 | 0.57 | 1.36 | 0.93 | 0.066 | 0.018 | 70.1 |
| RS | -295 | 0.21 | 1.87 | 9.11 | 1.62 | 10.6 | 61.2 | 28.3 | 4.5 |  | 12.98 | 0.21 | 0.64 | 1.56 | 1.00 | 0.067 | 0.022 | 68.3 |
| RS | -300 | 0.24 | 2.19 | 9.20 | 2.34 | 9.4 | 59.4 | 31.2 | 4.6 |  | 12.48 | 0.21 | 0.61 | 1.39 | 0.99 | 0.060 | 0.021 | 69.0 |
